# Right ventricular insertion site fibrosis in a dilated cardiomyopathy referral population: phenotypic associations and value for the prediction of heart failure admission or death

**DOI:** 10.1186/s12968-021-00761-0

**Published:** 2021-06-17

**Authors:** Yoko Mikami, Aidan Cornhill, Steven Dykstra, Alessandro Satriano, Reis Hansen, Jacqueline Flewitt, Michelle Seib, Sandra Rivest, Rosa Sandonato, Carmen P. Lydell, Andrew G. Howarth, Bobak Heydari, Naeem Merchant, Nowell Fine, James A. White

**Affiliations:** 1 Stephenson Cardiac Imaging Centre, Libin Cardiovascular Institute of Alberta, University of Calgary, #0700, SSB, Foothills Medical Centre, 1403-29th St. NW, Calgary, AB T2N2T9 Canada; 2 Department of Diagnostic Imaging, Cumming School of Medicine, University of Calgary, Calgary, AB Canada; 3 Department of Cardiac Sciences, Cumming School of Medicine, University of Calgary, Calgary, AB Canada

**Keywords:** Cardiovascular magnetic resonance, Cardiomyopathy, Heart failure, Remodeling, Prognosis

## Abstract

**Background:**

Dilated cardiomyopathy (DCM) is increasingly recognized as a heterogenous disease with distinct phenotypes on late gadolinium enhancement (LGE) cardiovascular magnetic resonance (CMR) imaging. While mid-wall striae (MWS) fibrosis is a widely recognized phenotypic risk marker, other fibrosis patterns are prevalent but poorly defined. Right ventricular (RV) insertion (RVI) site fibrosis is commonly seen, but without objective criteria has been considered a non-specific finding. In this study we developed objective criteria for RVI fibrosis and studied its clinical relevance in a large cohort of patients with DCM.

**Methods:**

We prospectively enrolled 645 DCM patients referred for LGE-CMR. All underwent standardized imaging protocols and baseline health evaluations. LGE images were blindly scored using objective criteria, inclusive of RVI site and MWS fibrosis. Associations between LGE patterns and CMR-based markers of adverse chamber remodeling were evaluated. Independent associations of LGE fibrosis patterns with the primary composite clinical outcome of heart failure admission or death were determined by multivariable analysis.

**Results:**

The mean age was 56 **±** 14 (28% female) with a mean left ventricular (LV) ejection fraction (LVEF) of 37%. At a median of 1061 days, 129 patients (20%) experienced the primary outcome. Any abnormal LGE was present in 306 patients (47%), inclusive of 274 (42%) meeting criteria for RVI site fibrosis and 167 (26%) for MWS fibrosis. All with MWS fibrosis showed RVI site fibrosis. Solitary RVI site fibrosis was associated with higher bi-ventricular volumes [LV end-systolic volume index (78 ± 39 vs. 66 ± 33 ml/m^2^, p = 0.01), RV end-diastolic volume index (94 ± 28 vs. 84 ± 22 ml/m^2^ (p < 0.01), RV end-systolic volume index (56 ± 26 vs. 45 ± 17 ml/m^2^, p < 0.01)], lower bi-ventricular function [LVEF 35 ± 12 vs. 39 ± 10% (p < 0.01), RV ejection fraction (RVEF) 43 ± 12 vs. 48 ± 10% (p < 0.01)], and higher extracellular volume (ECV). Patient with solitary RVI site fibrosis experienced a non-significant 1.4-fold risk of the primary outcome, increasing to a significant 2.6-fold risk when accompanied by MWS fibrosis.

**Conclusions:**

RVI site fibrosis in the absence of MWS fibrosis is associated with bi-ventricular remodelling and intermediate risk of heart failure admission or death. Our study findings suggest RVI site fibrosis to be pre-requisite for the incremental development of MWS fibrosis, a more advanced phenotype associated with greater LV remodeling and risk of clinical events.

**Supplementary Information:**

The online version contains supplementary material available at 10.1186/s12968-021-00761-0.

## Background

Patients with dilated cardiomyopathy (DCM) are recognized to have a significant prevalence of replacement myocardial fibrosis as identified by late gadolinium enhancement (LGE) cardiovascular magnetic resonance (CMR) [1–4]. Studies performed in this referral population have identified strong prognostic value for its presence, particular for the mid-wall septal striae (MWS) pattern of fibrosis that is typically observed in 25–30% of referral patients [1, 4, 5]. This pattern is believed to reflect an advanced state of adverse remodelling and has been associated with elevated risk of cardiac mortality, sudden cardiac death (SCD) or appropriate implantable cardioverter-defibrillator (ICD) therapy [1, 2, 4–7]. Prevalence of other patterns of fibrosis have similarly been explored and demonstrated that having multiple fibrosis patterns is associated with incremental risk [6]. However, despite reported as the most commonly observed pattern [2], right ventricular (RV) insertion (RVI) site fibrosis has been least studied [8, 9], lacks objective criteria, and is of uncertain relevance to clinical practice.

The RV pedicles are recognized to experience elevated wall tension during states that contribute to RV volume and/or pressure overload [10]. Cohort studies in relevant referral populations, such as pulmonary artery hypertension and tetralogy of Fallot, have convincingly shown that replacement fibrosis occurs at high frequency where the RV pedicles insert into the septal LV myocardium, and that this may herald adverse outcomes [11–15]. DCM is increasingly being recognized as a poly-phenotypic disease that may affect multiple cardiac chambers, inclusive of the RV. Expanding literature supports that DCM commonly demonstrates bi-ventricular involvement and that markers of RV remodelling and contractile performance may provide similar importance to those of its left-sided counterpart [16–18]. Given this, interest in RVI site fibrosis as a marker of adverse remodelling in this population appears justified. To date, only one study has focussed on exploring the prevalence and prognostic relevance of this marker in a DCM referral population, concluding an intermediate but non-significant elevation in risk for major cardiovascular events [8]. However, the prevalence of this marker relative to MWS fibrosis, and their respective influence on bi-ventricular remodelling and clinical outcomes has not been explored to date.

We hypothesized that RVI site fibrosis reflects an adaptive expansion of extracellular matrix at the RV pedicles in response to altered RV loading conditions that are experienced during the development of DCM. We further hypothesized that this marker may herald more advanced stages of remodelling associated with greater likelihood of heart failure-related events. In a large, prospectively recruited cohort of 645 patients referred to LGE-CMR for DCM we examined the prevalence and associated phenotypic features of RVI site fibrosis and MWS fibrosis, inclusive of their associations with bi-ventricular chamber volumetry, function and left ventricular (LV) extracellular volume (ECV) fraction. Their respective influence on the future occurrence of heart failure admission or death was also explored.

## Methods

### Study design and population

This was a prospective observational cohort study of subjects recruited between January 2015 and May 2018 and followed for a minimum of 12-months. The study was a pre-defined sub-cohort analysis of the Cardiovascular Imaging Registry of Calgary (CIROC) at the Libin Cardiovascular Institute of Alberta (NCT04367220). All patients in Southern Alberta referred for CMR imaging are recruited by, and data collected using commercial software (cardioDI™, Cohesic Inc., Calgary, Canada). This is inclusive of tablet-based patient engagement and consent, deployment of standardized patient-reported health questionnaires, indication-based CMR protocolling, standardized test reporting and phenotype coding, and automated linkage to electronic health record data inclusive of laboratory test results and administratively coded outcomes.

Patients were considered eligible for this study based on the following criteria; (i) referral for CMR-based evaluation of DCM: (ii) confirmation of LV ejection fraction (LVEF) ≤ 50% by CMR-based quantification, (iii) no prior clinical history of, or ICD-10 coded prior occurrence of myocardial infarction, percutaneous revascularization or coronary artery bypass grafting (CABG), and (iv) no LGE-based evidence of prior myocardial infarction, as defined by sub-endocardial pattern LGE in a typical coronary artery distribution. Patients with any clinically documented history of pulmonary arterial hypertension, congenital heart disease, severe valvular insufficiency/stenosis, or other cardiomyopathy diagnosis were excluded. Specifically, other excluded cardiomyopathy diagnoses included: acute myocarditis, cardiac sarcoidosis, cardiac amyloidosis, hypertrophic cardiomyopathy, arrhythmogenic right ventricular cardiomyopathy (ARVC), restrictive cardiomyopathy, or constrictive pericardial disease. Only patients completing at least 12-months of clinical follow-up were considered eligible. A post-hoc sub-study was performed with stratification of the referral population to those meeting objective dilation criteria, according to the consensus guideline for a body surface area (BSA)-indexed LV end-diastolic volume (LVEDVI) [19].

The study was approved the Conjoint Health Research Ethics Board at University of Calgary and all subjects provided written informed consent. All research activities were performed in accordance with the Declaration of Helsinki.

### CMR imaging and analysis protocol

CMR imaging was performed using 3T clinical CMR scanners (Prisma or Skyra, Siemens Healthineers, Erlangen, Germany). Standardized CMR imaging protocols were used routinely inclusive of cine imaging using a balanced steady-state free precession (bSSFP) pulse sequence and LGE imaging using a standard inversion recovery gradient echo pulse sequence both performed in standard 2, 3, and 4-chamber long axis views and sequential short-axis slices. LGE imaging was performed 10-min following intravenous administration of gadolinium contrast (0.1 mmol/kg; Gadovist; Bayer Healthcare, Berlin, Germany). Typical imaging parameters were: 6 mm slice thickness, 2 mm gap, TE 3.9 ms, flip angle 20°, matrix 256 × 205, segments 13–21, iPAT 2. T1 mapping was performed using a modified Look-Locker inversion recovery (MOLLI) pulse sequence in basal and mid-level short axis views prior to, and 15-min following intravenous contrast injection.

Quantitative image analysis was performed using commercially available software (cvi42; Circle Cardiovascular Imaging Inc., Calgary, Alberta, Canada) and with use of standardized operational procedures adherent to published Society for Cardiovascular Magnetic Resonance (SCMR) recommendations [20]. Short axis cine images were analyzed using semi-automated contour tracing of endocardial and epicardial borders to obtain the LV end-diastolic volume (LVEDV), LV end-systolic volume (LVESV), LVEF, LV mass, RV end-diastolic volume (RVEDV), RV end-systolic volume (RVESV), and RV ejection fraction (RVEF), as previously described [18]. For the LV, papillary muscles were included in the LV mass. Left atrial (LA) volumes were measured at the LV end-systolic phase prior to mitral valve opening using the bi-plane area-length method from temporally matched 4- and 2-chamber cine views. All volumetric analyses were indexed to BSA, where appropriate, using the Mosteller formula.

**Fig. 1:**
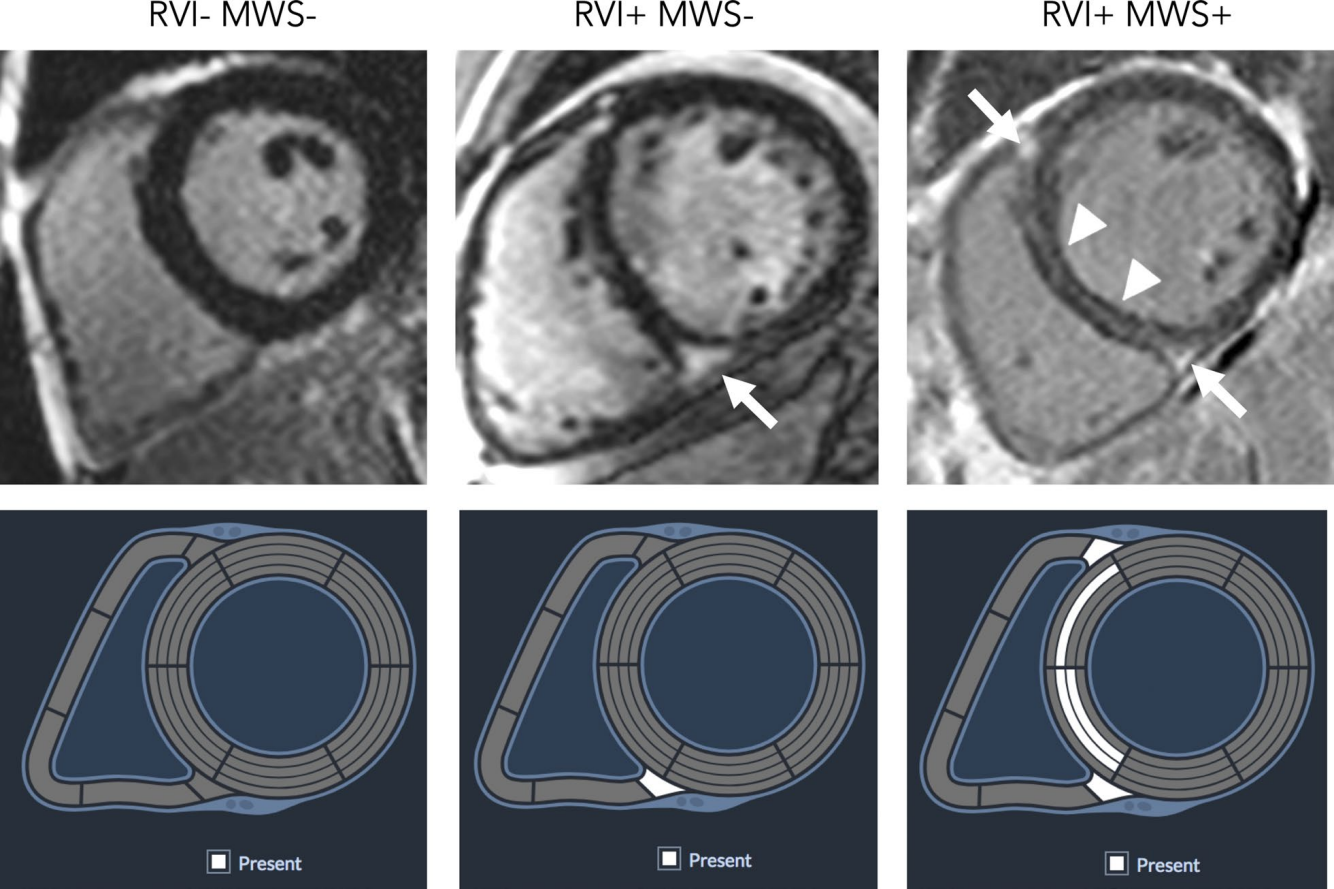
Example late gadolinium enhancement (LGE) images for: Left: Patient with neither right ventricular insertion site (RVI) fibrosis nor mid-wall septal striae (MWS) fibrosis, Middle: RVI fibrosis alone (arrow), and Right: both RVI and MWS fibrosis (RVI fibrosis with arrows, MWS fibrosis with arrowheads). Lower panel shows corresponding scoring of LGE patterns using standardized reporting architecture

**Fig. 2:**
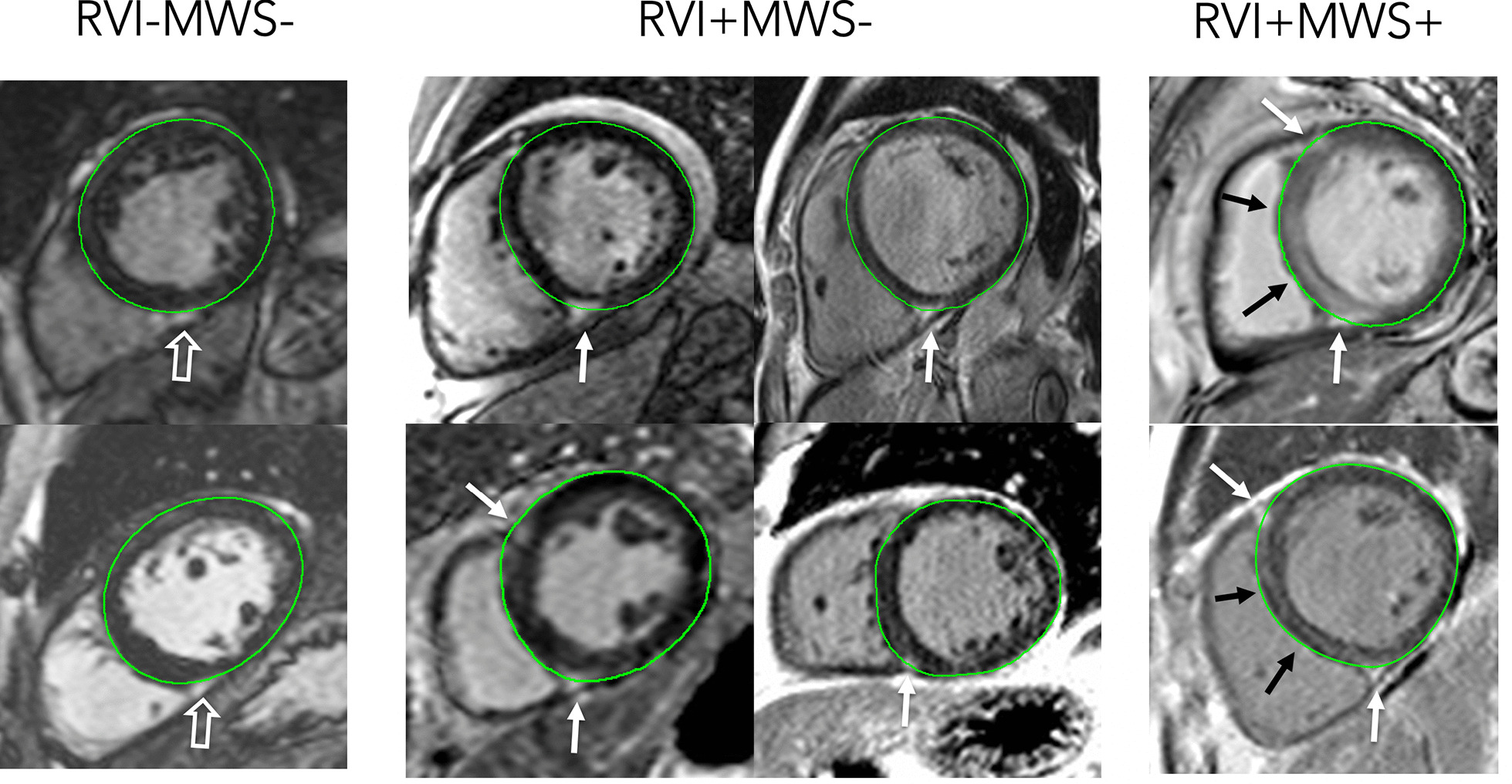
Example late gadolinium enhancement (LGE) images of patients with and without right ventricular insertion (RVI) and mid-wall striae (MWS) patterns of fibrosis. Left: Examples of suspected RVI fibrosis not meeting objective criteria. Middle: Examples of RVI fibrosis meeting objective criteria (signal extending through rounded epicardial contour). Right: Examples of combined RVI and MWS fibrosis

A standardized reporting interface (cardioDI™, Cohesic Inc, Calgary, Alberta, Canada) was used to code the presence, location and extent for all observed patterns of replacement myocardial fibrosis (Fig. 1). Patterns of fibrosis were classified as subendocardial, mid-wall septal striae, RVI site, mid-wall patchy, subepicardial, and diffuse global, as previously described [2]. All LGE coding was performed by experienced readers with blinded adjudication by a core laboratory reader (YM). For disagreement in pattern scoring the study was reviewed by a third expert reader (JW) to provide a final consensus coding. The spatial distribution and extent of fibrosis was coded for each of the American Heart Association (AHA) segments [21] using a sub-segmental model, each segment divided into four zones, as previously validated [22]. Objective criteria were used to define RVI site fibrosis, this requiring the presence of intra-myocardial signal enhancement extending from an RV pedicle through a rounded epicardial contour of the LV epicardial border into the LV myocardium, as shown in Fig. 2. The reporting of MWS fibrosis required the visual presence of a linear hyper-intensity of greatest intensity at the mid-myocardial region of the interventricular septum, as shown in Fig. 1. These patterns were required to be visualized on at least two contiguous or perpendicular views. Other visually identified fibrosis patterns were coded as mid-wall patchy, sub-epicardial or diffuse, as previously described [2].

**Fig. 3:**
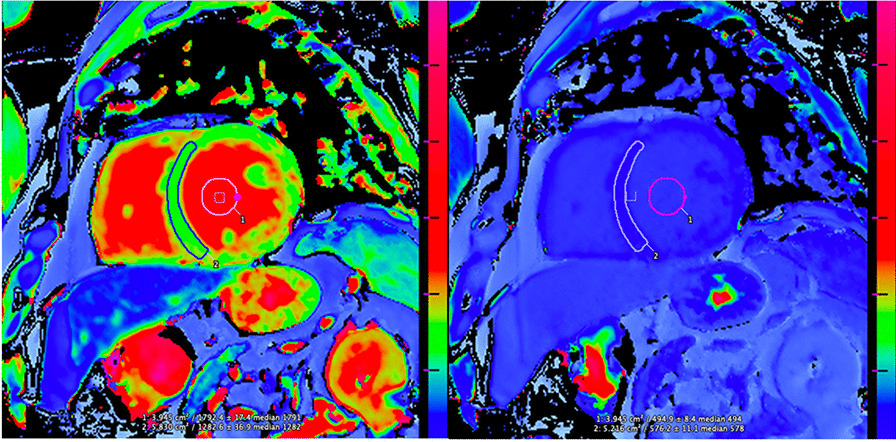
Example of short axis, mid-ventricular T1 mapping using modified Look-Locker inversion recovery (MOLLI) sequence with manual region of interest-based quantification of native T1 (left) and post-contrast T1 (right) for both myocardium and blood pool

Pre- and post-contrast T1 mapping was evaluated to estimate the ECV of mid ventricular tissue. Regions of interests were drawn in the myocardium and blood pool of mid-ventricular short-axis image, as shown in Fig. 3. ECV was calculated by the following formula [23]:

$$ECV=(1-hematocrit)(1/{T1_{myo\_post}}-1/{T1_{myo\_pre}})/(1/{T1_{blood\_post}}-1/{T1_{blood\_pre}})$$

where T1_myo_pre_ and T1_myo_post_ are native and post-contrast measures of myocardial T1, and T1_blood_pre_ and T1_blood_post_ are native and post-contrast measures of T1 in the blood pool.

### Clinical outcome collection and adjudication

Clinical outcomes were collected by way of administrative data matching followed by adjudication of all identified events through medical chart review. Heart failure admission was defined according to occurrence of an ICD-10 coded heart failure admission (154.X) in conjunction with hospital admission ≥ 24 h duration. All-cause mortality was determined by combined consideration of data matching from Vital Statistics Alberta and In-hospital coded deaths. No patients were lost to follow-up.

### Statistical analysis

All descriptive statistics were expressed as mean ± standard deviation (SD) and categorical variables were expressed in counts with percentages. Comparison between two groups was performed with the independent Student t-test or Mann–Whitney U test, comparison between three groups was performed using ANOVA or Kruskal–Wallis test depending on the distribution. Pairwise comparison with Bonferroni procedure was performed as post-hoc analysis for three group comparison. Fisher exact test was used to compare categorical data. Missing variables, when identified, were specified in Table 1 and subjects with missing data points excluded from comparison for respective variables.

**Table 1:** Baseline clinical and CMR characteristics for patients with and without occurrence of the primary outcome

Characteristics	Total cohort (N = 645)	Event− (N = 516)	Event+ (N = 129)	HR (95% CI)	p value
Age (years)	56 ± 14	55 ± 14	58 ± 15	1.02 (1.002–1.03)	0.03*
Female, n (%)	180 (28)	140 (27)	40 (31)	1.20 (0.83–1.74)	0.3
Systolic blood pressure (mmHg)	116 ± 18	117 ± 17	113 ± 20	0.99 (0.98–0.997)	0.01*
Diastolic blood pressure (mmHg)	70 ± 13	71 ± 13	68 ± 13	0.99 (0.97–1.002)	0.09
Heart rate (bpm)	71 ± 16	70 ± 15	75 ± 17	1.02 (1.007–1.03)	0.001*
BMI (kg/m^2^)	29 ± 6	29 ± 6	30 ± 8	1.02 (0.99–1.05)	0.15
Hypertension, n (%)	228 (35)	173 (34)	55 (43)	1.41 (0.99–1.99)	0.06
Diabetes, n (%)	128 (20)	85 (17)	43 (33)	2.33 (1.62–3.37)	< 0.001*
Hyperlipidemia, n (%)	343 (53)	259 (50)	84 (65)	1.74 (1.21–2.51)	0.003*
Smoking, n (%)	120 (20)	96 (20)	24 (21)	1.07 (0.68–1.67)	0.78
Creatinine (µmol/l)	95 ± 46	94 ± 49	100 ± 32	1.00 (0.999- 1.00)	0.16
NYHA class III or IV, n (%)	153 (26)	112 (24)	41 (37)	1.81 (1.23–2.66)	0.002*
Medications					
ACE-I or ARB, n (%)	524 (81)	407 (79)	117 (91)	2.41 (1.33–4.36)	0.004*
Beta blocker, n (%)	530 (82)	413 (80)	117 (91)	2.20 (1.21–3.98)	0.009*
Diuretics, n (%)	331 (51)	234 (45)	97 (75)	3.28 (2.2–4.90)	< 0.001*
Digoxin, n (%)	56 (9)	38 (7)	18 (14)	1.9 (1.15–3.13)	0.01*
Statin, n (%)	253 (39)	193 (37)	60 (47)	1.41 (0.999–1.997)	0.05
CCB, n (%)	76 (12)	58 (11)	18 (14)	1.23 (0.75–2.02)	0.42
Amiodarone, n (%)	31 (5)	19 (4)	12 (9)	2.13 (1.18–3.86)	0.01*
CMR-Non LGE variables					
LVEDVI (ml/m^2^)	113 ± 38	110 ± 36	126 ± 45	1.01 (1.005–1.01)	< 0.001*
LVESVI (ml/m^2^)	74 ± 37	70 ± 34	89 ± 43	1.01 (1.006–1.01)	< 0.001*
LVEF (%)	37 ± 11	38 ± 10	32 ± 12	0.95 (0.94–0.97)	< 0.001*
LV mass index (g/m^2^)	70 ± 22	68 ± 20	78 ± 25	1.02 (1.01–1.02)	< 0.001*
RVEDVI (ml/m^2^)	87 ± 25	87 ± 23	89 ± 31	1.003 (0.996–1.01)	0.36
RVESVI (ml/m^2^)	49 ± 22	47 ± 19	54 ± 28	1.01 (1.01–1.02)	< 0.001*
RVEF (%)	46 ± 12	47 ± 11	42 ± 13	0.97 (0.95–0.98)	< 0.001*
LA volume index (ml/m^2^)	43 ± 18	42 ± 17	48 ± 20	1.02 (1.01–1.02)	< 0.001*
CMR-LGE variables					
RV insertion site	274 (42)	198 (38)	76 (59)	2.11 (1.48–2.99)	< 0.001*
Mid-wall striae	167 (26)	112 (22)	55 (43)	2.33 (1.64–3.31)	< 0.001*
Mid-wall patchy	10 (2)	8 (2)	2 (2)	1.01 (0.25–4.08)	0.99
Sub-epicardial	54 (8)	45 (9)	9 (7)	0.82 (0.42–1.62)	0.57
Diffuse	1 (0.2)	0 (0)	1(0.8)	–	–
Any LGE	306 (47)	227 (44)	79 (61)	1.88 (1.32–2.68)	< 0.001*

Univariable hazards provided. HR data were available for 644 patients, Smoking for 593 patients, creatinine for 493 patients, NYHA for 584 patients, LV mass index for 640 patients, RVEDVI for 643 patients, RVESVI and RVEF for 642 patients and LA max indexed for 635 patients

*EDVI* End-diastolic volume indexed to body surface area, *EF* ejection fraction, *ESVI* end-systolic volume indexed to body surface area, *LA* left atrial, *LGE* late gadolinium enhancement, *LV* left ventricular, *NYHA* New York Heart Association, *RV* right ventricular

Continuous data are expressed as mean ± SD, categorical data as n (%). *p < 0.05

Univariable associations between demographic or CMR characteristics and the outcomes were performed using Cox proportional hazards regression. Multivariable Cox regression analysis was performed to assess associations between with and without the most prevalent patterns of LGE, these being RVI site fibrosis or MWS fibrosis, and the outcome. Covariates with a p-value < 0.1 were considered eligible for adjustment within each multivariable model. All models were assessed for collinearity and proportional hazards assumption.

Intra- and inter-observer reliability was tested using 30 randomly selected studies stratified to achieve a balanced representation of normal, RVI site positive and MWS pattern positive cases. Two observers scored all studies, followed by repeat scoring of cases in random order by the second observer. Agreement was described by Cohen’s kappa coefficient.

Statistical analyses were performed using RStudio 1.2.5019 (R Foundation for Statistical Computing, Vienna, Austria) and SPSS for Macintosh (version 24.0, Statistical Package for the Social Sciences, International Business Machines, Inc., Armonk, New York, USA).

## Results

### Baseline patient characteristics

Six hundred and forty-five patients were consecutively recruited and met study eligibility criteria. Baseline clinical characteristics for all subjects, as well as those with and without the primary outcome are provided in Table 1. The mean age was 56 ± 14 years with 180 (28%) being female. Two-hundred and twenty-eight patients (35%) had history of hypertension, and 128 patients (20%) were diabetic.

The mean LVEF was 37 ± 11%, and the mean RVEF was 46 ± 12%. All additional CMR-based chamber measurements are summarized in Table 1.

### Prevalence of RVI site fibrosis versus other patterns of fibrosis

Replacement myocardial fibrosis was observed in 306 (47%) patients (Table 1). This included 274 patients (42%) meeting objective criteria for RVI site fibrosis and 167 (26%) patients meeting criteria for MWS fibrosis. Importantly, based upon objective criteria, RVI site fibrosis was present among all subjects meeting criteria for MWS fibrosis. Other patterns of non-ischemic fibrosis were identified with the following prevalence: 54 (8.4%) with sub-epicardial fibrosis (25 isolated and 29 combined with another pattern), 10 (1.6%) mid-wall patchy fibrosis (6 isolated and 4 combined with another pattern) and 1 with isolated diffuse fibrosis.

Among patients with RVI site but no MWS fibrosis (RVI+MWS−, n = 107) secondary patterns of fibrosis were observed in 9 (8%): 6 sub-epicardial and 3 mid-wall patchy fibrosis. Patients with combined RVI and MWS fibrosis (RVI+MWS+, n = 167) showed secondary patterns in 24 (14%): 23 sub-epicardial and 1 mid-wall patchy fibrosis.

### RVI site fibrosis: associations with chamber remodeling and contractile function

As shown in Additional file 1: Table S1, patients with RVI fibrosis had significantly higher RVEDVI (92 ± 27 vs. 84 ± 22 ml/m^2^ without RVI fibrosis; p < 0.01), higher RVESVI (54 ± 26 vs. 45 ± 17 ml/m^2^, p < 0.01), and lower RVEF (43 ± 13 vs. 48 ± 10%, p < 0.01). Similarly, these subjects showed higher LVEDVI (123 ± 40 vs. 106 ± 36 ml/m^2^, p < 0.01), higher LVESVI (85 ± 39 vs. 67 ± 33 ml/m^2^, p < 0.01) and lower LVEF (33 ± 11 vs. 39 ± 10%, p < 0.01). LA volumes were significantly higher among patients with RVI site fibrosis (46 ± 19 vs. 40 ± 16 ml/m^2^ without RVI site fibrosis, p < 0.01).

**Fig. 4:**
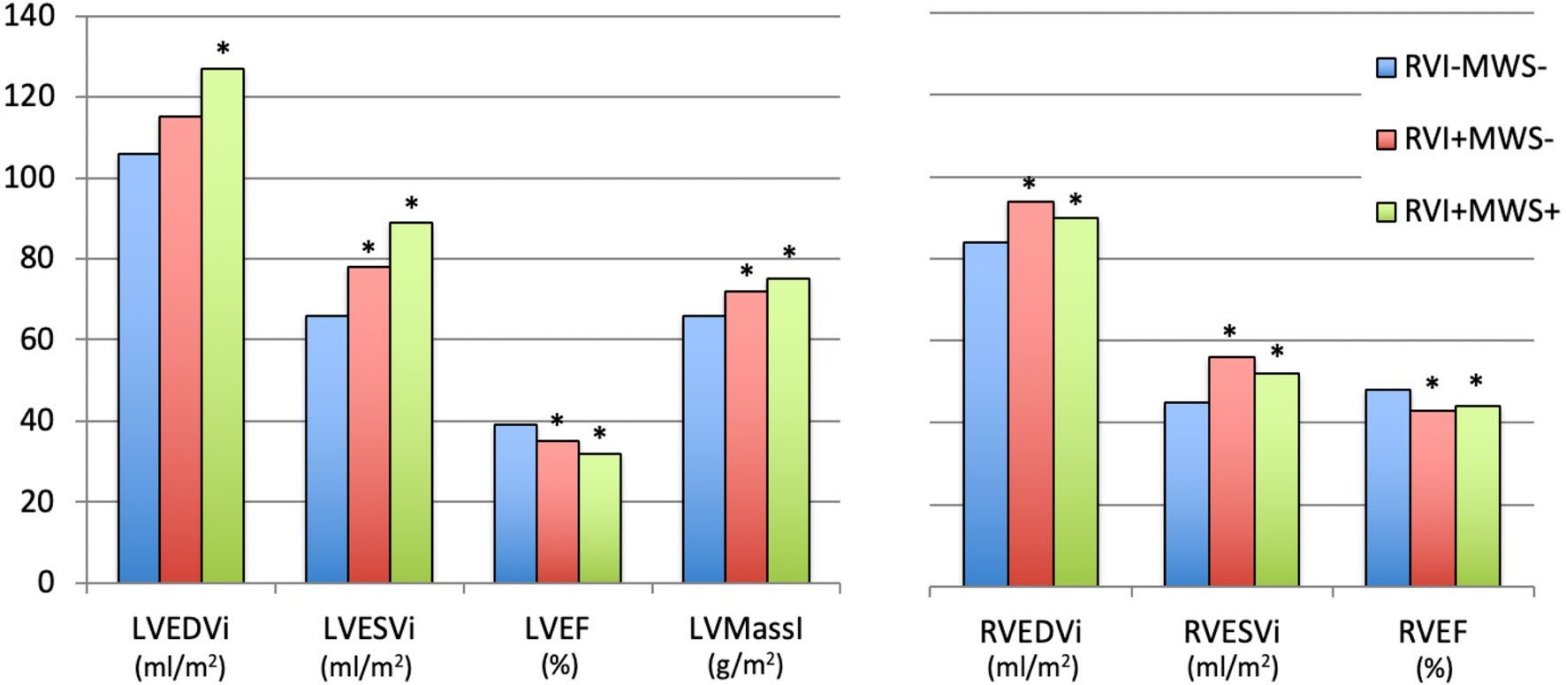
Relationships observed between patient fibrosis phenotype versus volumetric measures of left ventriuclar and right ventricular remodeling and contractile dysfunction. Patients with RVI fibrosis showed greater bi-ventricular volumes with lower bi-ventricular function. *p < 0.05 versus RVI-MWS-group

Sub-stratification of patients into groups with RVI fibrosis alone versus those with RVI plus MWS was subsequently performed, as shown in Fig. 4. Bi-ventricular markers of remodeling and ejection fraction are displayed for the following three groups: no RVI or MWS fibrosis (RVI−MWS−), RVI fibrosis alone (RVI+MWS−), and both RVI and MWS patterns (RVI+MWS+). Versus RVI−MWS−, patients with isolated RVI site fibrosis showed significantly higher bi-ventricular volumes and reduced ejection fractions, as follows; LVESVI (78 ± 39 vs. 66 ± 33 ml/m^2^, p = 0.01); LVEF (35 ± 12 vs. 39 ± 10%, p < 0.01); RVEDVI (94 ± 28 vs. 84 ± 22 ml/m^2^, p < 0.01); RVESVI (56 ± 26 vs. 45 ± 17 ml/m^2^, p < 0.01); RVEF (43 ± 12 vs. 48 ± 10%, p < 0.01).

### RVI site fibrosis: associations with extracellular volume (ECV) fraction

A sub-group of 80 subjects underwent parametric T1 mapping to identify associations between the presence of RVI site fibrosis and LV myocardial ECV. Subject selection for this analysis was based upon availability of high-quality T1-mapping image datasets with prevalence of respective phenotypes as follows; RVI−MWS− (n = 34), RVI+MWS− (n = 19), and RVI+MWS+ (n = 27). ECV was lowest among RVI−MWS− subjects with a mean ECV of 31 **±** 4% (laboratory healthy reference value: 26 ± 1.9%). By comparison, patients with isolated RVI fibrosis (RVI+MWS−) or combined pattern fibrosis (RVI+MWS+) showed significant elevation in mid-ventricular ECV (34 ± 6% and 33 ± 5%, respectively [ANOVA p = 0.03]).

### RVI site fibrosis: association with the primary outcome

During a median follow up of 1061 days (IQR = 674 days), 129 patients (20%) experienced the primary composite outcome of heart failure hospitalization or death (108 heart failure hospitalizations, 21 deaths). Univariable associations between baseline characteristics and the primary outcome are shown in Table 1. The following variables were significant predictors; age at scan, systolic blood pressure, heart rate, diabetes, hyperlipidemia, New York Heart Association (NYHA) class III or IV, use of angiotensin converting enzyme inhibitor (ACEi) or angiotensin II receptor blocker (ARB), beta blocker, diuretics, digoxin, amiodarone, LVEDVI, LVESVI, LVEF, LV mass index, RVESVI, RVEF, indexed LA volume, any RVI site fibrosis, MWS fibrosis, and any LGE. Patients with RVI site fibrosis with or without MWS (n = 274, 42%) experienced a cumulative event rate of 28% (76 events) versus 14% (53 events) for patients without RVI site fibrosis. The unadjusted hazard of this marker for the primary outcome was 2.11 (95% CI 1.48–2.99, p < 0.001). By comparison, patients with a MWS fibrosis pattern (n = 167, 26%), experienced a cumulative event rate of 33% (55 events) versus 15% (74 events) for patients without MWS fibrosis (HR 2.33, 95% CI 1.64–3.31, p < 0.001).

RVI fibrosis (with or without MWS) remained an independent predictor of the primary outcome in three different multivariable models, these designed to provide robust adjustment for patient comorbidities (Model 1), medication use (Model 2), and balanced adjustment of both (Model 3), as shown in Table 2. Model 1 identified any RVI fibrosis to have an adjusted HR of 1.75 (95% CI 1.18–2.60 p = 0.01) following adjustment for age, systolic blood pressure, heart rate, hypertension, diabetes, hyperlipidemia, NYHA III or IV, LVEF, RVEF and indexed LA volume. Model 2 provided a HR of 1.66 (95% CI 1.12–2.46, p = 0.01) following adjustment for age, systolic blood pressure, heart rate, NYHA III or IV, ACE/ARB, beta blocker, diuretics, LVEF, RVEF, and indexed LA volume. Model 3 provided a HR of 1.61 (95% CI 1.09–2.40, p = 0.02) following adjustment for age, hypertension, diabetes, NYHA III or IV, ACE/ARB, beta blocker, diuretics, LVEF, RVEF, and indexed LA volume.

**Table 2:** Multivariable models inclusive of baseline characteristics and RVI fibrosis for the primary outcome

Characteristics	Univariable			Model 1^a^			Model 2^b^			Model 3^c^		
	HR	95% CI	p value	HR	95% CI	p value	HR	95% CI	p value	HR	95% CI	p value
Demographics												
Age	1.02	1.002–1.03	0.03									
Systolic blood pressure	0.99	0.98–0.997	0.01									
Heart rate	1.02	1.007–1.03	0.001	1.02	1.004–1.03	0.01	1.02	1.003–1.03	0.01			
Hypertension	1.41	0.99–1.99	0.06									
Diabetes	2.33	1.62–3.37	< 0.001	2.32	1.57–3.43	< 0.001				1.97	1.32–2.94	0.001
Hyperlipidemia	1.74	1.21–2.51	0.003									
NYHA 3–4	1.81	1.23–2.66	0.002									
Medications												
ACEi/ARB	2.41	1.33–4.36	0.004									
Beta blocker	2.20	1.21–3.98	0.009									
Diuretics	3.28	2.2–4.90	< 0.001				2.18	1.37–3.47	0.001	1.84	1.14–2.96	0.01
CMR variables												
LVEF	0.95	0.94–0.97	< 0.001	0.97	0.95–0.98	< 0.001	0.98	0.96–0.995	0.01	0.97	0.95–0.99	0.001
RVEF	0.97	0.95–0.98	< 0.001									
LA volume index	1.02	1.01–1.02	< 0.001									
RVI fibrosis	2.11	1.48–2.99	< 0.001	1.75	1.18–2.60	0.01	1.66	1.12–2.46	0.01	1.61	1.09–2.40	0.02

*ACEi* Angiotensin-converting enzyme inhibitors, *ARB* Angiotensin II receptor blocker, *BP* blood pressure, *EF* ejection fraction, *LA* left atrial, *LV* left ventricular, *NYHA* New York Heart Association, *RV* right ventricular, *RVI* right ventricular insertion point

^a^Model 1 represents multivariable Cox regression model with age, systolic BP, Heart rate, Hypertension, Diabetes, Hyperlipidemia, NYHA 3–4, LVEF, RVEF, indexed LA volume, RVI fibrosis (present or absent)

^b^Model 2 represents multivariable Cox regression model with age, systolic BP, Heart rate, NYHA 3–4, ACEi/ARB, Beta Blocker, Diuretics, LVEF, RVEF, indexed LA volume, RVI fibrosis (present or absent)

^c^Model 3 represents represents multivariable Cox regression model with age, Hypertension, Diabetes, NYHA 3–4, ACEi/ARB, Beta Blocker, Diuretics, LVEF, RVEF, indexed LA volume, RVI fibrosis (present or absent)

Identical multivariable analyses were performed replacing any RVI fibrosis with the less frequently observed combined pattern of RVI plus MWS fibrosis. This provided corresponding adjusted hazards of 2.02 (95% CI 1.36–3.00, p < 0.001) for Model 1, 1.93 (95% CI 1.31–2.85, p = 0.001) for Model 2, and 1.78 (95% CI 1.21–2.63, p = 0.004) for Model 3.

**Fig. 5:**
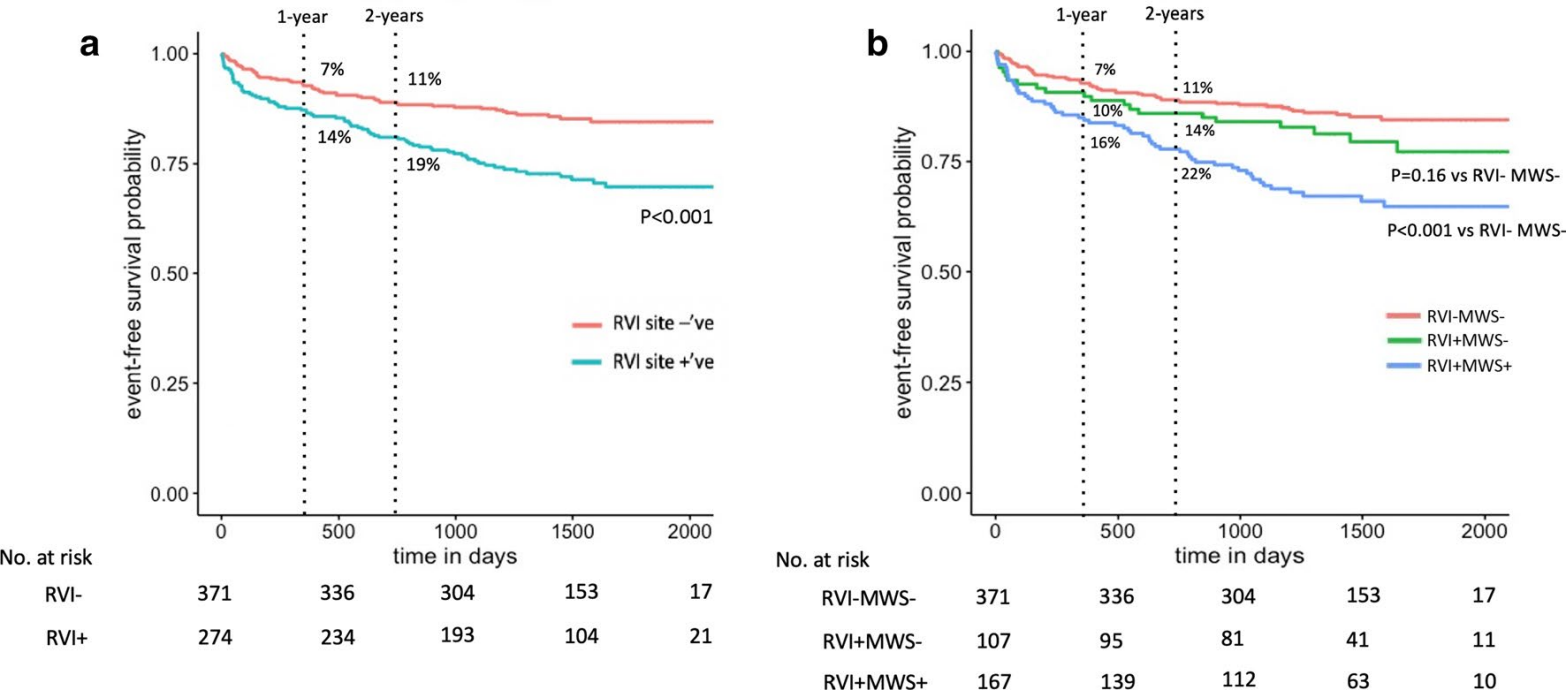
Kaplan–Meier analysis curves for the primary composite outcome. **a** Analysis according to presence or absence of RVI site fibrosis, demonstrating those with RVI site fibrosis had significantly worse event-free survival (p < 0.001). **b** Analysis stratified by the presence of RVI site fibrosis in isolation (RVI+MWS−) versus combined with MWS fibrosis (RVI+MWS+)

Kaplan–Meier analysis curves for patients with and without any RVI are shown in Fig. 5. Patients with RVI showed significantly worse event-free survival compared to those without (p < 0.001), with cumulative event rates of 14% (versus 7%) at 1-year, and 19% (versus 11%) at 2-years.

### RVI fibrosis alone vs. combined with MWS: associations with the primary outcome

**Fig. 6:**
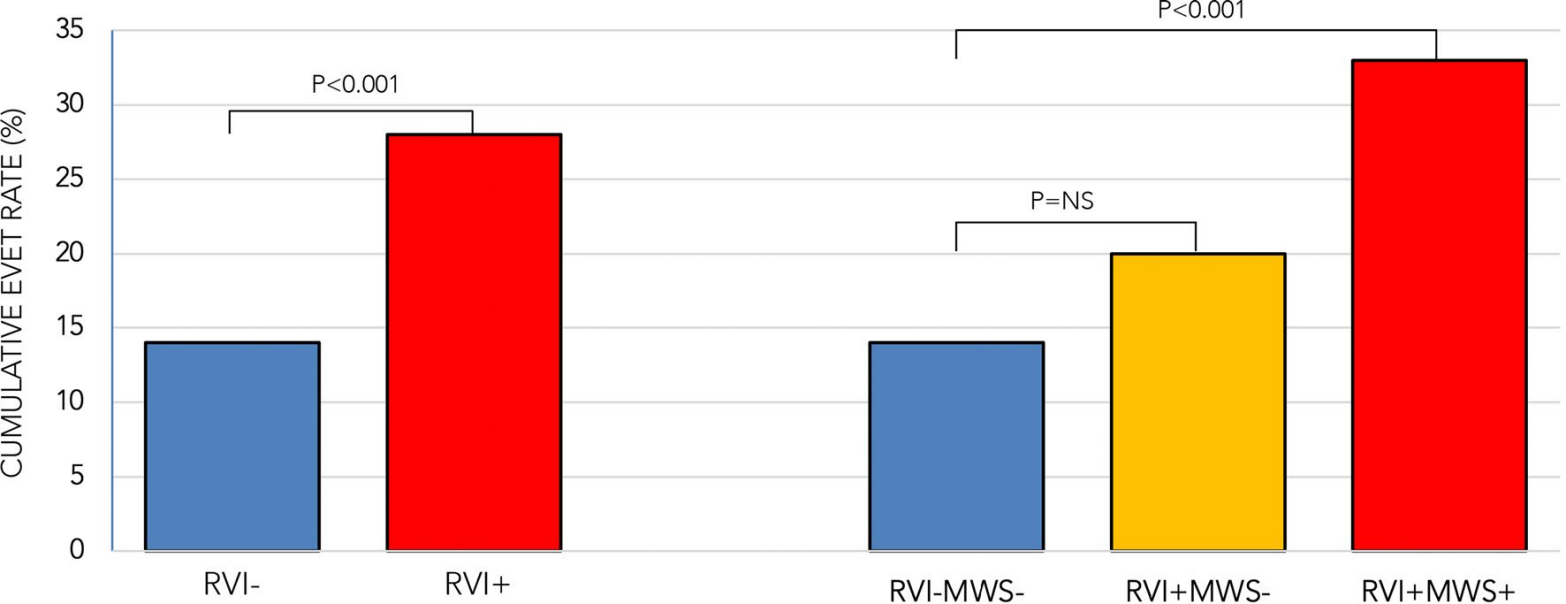
Comparison of cumulative event rates. Left: Patients with versus without RVI site fibrosis. Right: Sub-group analysis of RVI site positive patients stratified according to isolated presence (RVI+MWS−) or with incremental presence of MWS fibrosis (RVI+MWS+)

Exploratory subgroup analyses were undertaken to compare associations between solitary RVI fibrosis (RVI+MWS−, n = 107) and the combined RVI and MWS fibrosis pattern (RVI+MWS+, n = 167) with the primary outcome. As shown in Fig. 6, versus patients without fibrosis (RVI−MWS−, n = 371) who experienced 53 events (event rate 14%), patients with isolated RVI fibrosis experienced 21 events (20% event rate), and those with a combined pattern experienced 55 events (33% event rate). Versus patients without RVI fibrosis, isolated RVI site fibrosis was associated with a 1.4-fold increased risk for the primary outcome (HR 1.44, 95% CI 0.87–2.38, p = 0.16) while a combined RVI plus MWS pattern was associated with a 2.6-fold increased risk (95% CI 1.76–3.74, p < 0.001). Kaplan–Meier curves for each sub-group are provided in Fig. 5.

### Associations with the primary outcome according to LV dilation severity

Post-hoc analyses were conducted to explore the influence of LV dilation on the prognostic utility of RVI fibrosis in the referral population. Patients were stratified by BSA-indexed LVEDV according to standard deviation (SD) from sex-matched healthy reference values [19]. This resulted in three sub-groups: LVEDVI within 2SD (n = 294), LVEDVI 2-4SD (n = 162) and LVEDVI > 4SD (n = 189). Among these respective groups the primary outcome occurred in 40 (14%), 35 (22%), and 54 (29%) patients. The univariable hazards provided by any RVI site fibrosis for the primary outcome were 2.1 (95% CI 1.14–3.93, p = 0.02), 2.07 (95% CI 1.05–4.06, p = 0.04) and 1.50 (95% CI 0.85–2.64, p = 0.16), respectively. Identical post-hoc analysis was attempted following the incremental stratification of any RVI fibrosis into RVI+MWS− and RVI+MWS+ sub-phenotypes (referenced to RVI−MWS−). This exploratory analysis was under-powered but showed the RVI+MWS− sub-phenotype did not reach statistical significance for association with the primary outcome across dilation sub-groups. In contrast, the RVI + MWS + sub-phenotype maintained significant associations for all except severe dilation with respective univariable hazards of 2.56 (95% CI 1.23–5.30, p = 0.01), 3.06 (95% CI 1.52–6.17, p = 0.002) and 1.53 (95% CI 0.84–2.79, p = 0.17).

### Intra- and inter-observer reproducibility of RVI site and MWS fibrosis scoring

Good reproducibility of RVI site fibrosis was observed using the objective criteria. Respective kappa coefficients for intra- and inter-observer ratings were 0.93 and 0.88 respectively. By comparison, kappa coefficients for MWS fibrosis were lower at 0.91 and 0.82, respectively.

## Discussion

This prospective study was designed to explore the prevalence, phenotypic associations and prognostic significance of RVI site fibrosis in the context of MWS fibrosis for a DCM referral population. Using objective criteria, any RVI site fibrosis was identified in 42% of DCM patients, and was associated with increased bi-ventricular chamber volumes, reduced ejection fractions, and a 2.1-fold unadjusted risk of heart failure admission or death. This fibrosis pattern was observed both in isolation (39% of cases) and accompanied by MWS fibrosis (61% of cases). Isolated RVI site fibrosis was associated with objective markers of bi-ventricular remodeling and a non-significant 1.4-fold increased risk of heart failure admission or death. Specifically, this subgroup showed an 18% elevation in LVESVI, 4% absolute reduction in LVEF, 24% increase in RVESVI, 5% absolute reduction in RVEF, and 3% absolute elevation in mid septal ECV. Patients with incremental evidence of MWS fibrosis showed worsening of LV remodeling and a significant 2.3-fold risk of heart failure admission or death.

Our observations confirm that the RVI sites commonly experience a need to expand collagen content in the setting of DCM, and that this is associated with objective bi-ventricular remodeling and diffuse ECV expansion. While longitudinal studies are desirable, the ubiquitous presence of RVI site fibrosis in patients with MWS fibrosis strongly supports that its isolated presence represents an antecedent phenotype. As a requisite stage it is represented by objective bi-ventricular remodeling not yet sufficient to confer significantly elevated risk of heart failure admission or death. Therefore, while MWS fibrosis reflects an advanced clinical stage where major cardiovascular events are frequent [1, 2, 4–7], this again confirmed by our current study, observation of isolated RVI site fibrosis may identify patients with evolving remodeling that may uniquely benefit from targeted heart failure therapies.

Our findings are in agreement with the study previously reported by Yi, et al. that, in a detailed cohort study of 360 DCM patients undergoing CMR, showed isolated RVI site fibrosis to convey a non-significant trend towards elevated event risk [8]. Upon combination of this pattern with any other LV pattern, significant risk of future adverse events was then observed. This analysis did not assess the phenotypic associations or prognostic value of RVI site fibrosis versus MWS fibrosis. A recent study by Grigoratos, et al., performed in 420 clinically referred patients with apparently normal hearts identified that the presence of isolated RVI site fibrosis was not associated with future clinical events [9]. However, given its unique referral population and a lack of events (total 8), meaningful comparison to our current study is not possible.

The factors that contribute to the development of RVI site fibrosis in patients with DCM have not been defined and therefore remain speculative. However, meaningful insights can be gathered from prior studies performed across other disease states. Most notably, chronic elevations in RV systolic pressures have been associated with a higher prevalence of RVI site fibrosis in patients with pulmonary hypertension [11–13]. Similarly, chronic RV volume loading is recognized to be associated with this marker, particularly among those with surgically repaired tetralogy of Fallot [14, 15]. In these populations, the presence of RVI site fibrosis has been associated with adverse outcomes [13] or other surrogate markers of adverse outcomes [14].

Expanding evidence supports that patients with left-sided heart disease may similarly demonstrate irreversible remodeling of the RV myocardium [24, 25]. Nitsche, et al. recently studied 167 patients with heart failure and preserved ejection fraction (HFpEF) by CMR and right-heart catheterization. Significant associations between RVI site native T1 (a surrogate marker of interstitial fibrosis) were appreciated with pulmonary arterial wedge pressure, pulmonary arterial pressure and right atrial pressure [26]. T1 elevation was an independent predictor of death following adjustment for pulmonary arterial pressure, suggesting that RV afterload may not fully explain the pathophysiologic origin of RVI site fibrosis in this population. Patel et al. expanded on this work through a CMR-based study of 14 HFpEF patients with direct comparison to patients with pulmonary hypertension inclusive of their invasive hemodynamics. Despite significantly lower pulmonary arterial pressures, patients with HFpEF showed similar elevations in ECV of the RV free wall to those with pulmonary hypertension, as well as similar reductions in RVEF [27]. From these studies it appears that mechanisms other than transmissive hemodynamic loading may be contributory to adverse remodeling of the RV myocardium in patients with left-sided heart failure. However, studies inclusive of invasive hemodynamics are now required to confirm these relationships in the DCM population.

Compared to patients without identifiable RVI fibrosis (RVI−MWS−), patients with the RVI+MWS− and RVI+MWS+ phenotypes demonstrated incremental elevation in chamber volumes, reduction in chamber function, and elevation in mid ventricular ECV. The latter finding has been associated with higher mortality in patients with DCM [28], and provides validation for our objectively defined criteria used in this study to characterize pathologic forms of signal enhancement at the RVI site. It is important to stress routine use of objective criteria to ensure signal enhancement extends into the LV myocardium as RV blood pool signal may otherwise be commonly mistaken for this marker.

### Study limitations

Limitations of this study are recognized as patients were recruited from a single tertiary care institution. Accordingly, our results require confirmation using a multi-center study design. While our referral cohort was of substantial size, females were under-represented and sub-group analysis was limited by the number of events accrued for each sub-group. The latter is relevant to conclusions surrounding the prognostic significance of isolated RVI site fibrosis, where a non-significant 1.4-fold increased risk of the primary outcome (p = 0.16) was observed. Within a larger cohort study this may emerge to be significant. Further, through our detection of clinical events using administratively coded data, we may under-estimate event rates.

Our study focused on evaluating patients referred to CMR with a clinical diagnosis of DCM, this defined by criteria that did not mandate performance of invasive coronary angiography or stress perfusion testing. Accordingly, we cannot exclude the potential for sub-clinical ischemia in our population. Also, we did not exclude patients based on dilation criteria at time of CMR. To address this, we performed post-hoc analyses stratifying our referral population by CMR-based measures of LVEDVI. This demonstrated any RVI site fibrosis remained prognostic among patients with a LVEDVI within 2SD and between 2 and 4SD above CMR-based reference values. However, significance was lost for patients with severe (> 4SD) dilation. Accordingly, the incremental value of LGE-based phenotyping among patients with severe structural remodeling may be limited. We did not assess the impact of device-related therapy and, while excluding patients with known pulmonary hypertension, did not have consistent access to echocardiographic-based measures to exclude sub-clinical elevations in pulmonary arterial pressures. Finally, we acknowledge that the clinical outcome of heart failure admission can be influenced by regional practice patterns.

Our study design limits conclusions regarding sequential occurrence of, or mechanistic relationships between RVI site and MWS fibrosis patterns in patients with DCM. Longitudinal cohort studies inclusive of serial imaging are required to address such hypotheses. Histologic studies validating our objective criteria for RVI site fibrosis would also be of benefit, however, was beyond the scope of this study.

## Conclusions

Among patients referred for CMR-based evaluations of DCM, RVI site fibrosis (scored by objective criteria) was common and ubiquitously seen in patients with MWS fibrosis. Isolated RVI site fibrosis was associated with objective evidence of bi-ventricular remodeling. When employed as a solitary phenotypic risk marker, any RVI site fibrosis was associated with a 2.1-fold increased risk of heart failure or death. Incremental consideration of MWS fibrosis permits sub-classification into RVI+MWS− and RVI+MWS+ sub-groups, these respectively experiencing a non-significant 1.4-fold and significant 2.6-fold risk of the primary outcome versus RVI−MWS−.

Overall, our results offer support for RVI site fibrosis being an antecedent phenotypic stage to MWS fibrosis that is objectively associated with bi-ventricular remodeling and intermediate risk. Future studies may therefore be justified evaluating targeted therapeutic strategies to mitigate phenotype progression in patients with isolated RVI site fibrosis.

## Supplementary Information

**Additional file 1.** Baseline clinical and CMR characteristics for patients with and without right ventricular insertion (RVI) site fibrosis.

## Data Availability

The data sets used and/or analyzed during the current study are available from the corresponding author on reasonable request.

## Abbreviations

ACEi: Angiotensin converting enzyme inhibitor
AHA: American Heart Association
ARB: Angiotensin II receptor blocker
ARVC: Arrhythmogenic right ventricular cardiomyopathy
BSA: Body surface area
bSSFP: Balanced steady state free precession
CABG: Coronary artery bypass grafting
CMR: Cardiovascular magnetic resonance
DCM: Dilated cardiomyopathy
ECV: Extracellular volume
HFpEF: Heart failure with preserved ejection fraction
ICD: Implantable cardioverter defibrillator
LA: Left atrium/left atrial
LGE: Late gadolinium enhancement
LV: Left ventricle/left ventricular
LVEDV: Left ventricular end-diastolic volume
LVEDVI: Left ventricular end-diastolic volume index
LVEF: Left ventricular ejection fraction
LVESV: Left ventricular end-systolic volume
LVESVI: Left ventricular end-systolic volume index
MOLLI: Modified Look-Locker inversion recovery
MWS: Mid wall striae
NYHA: New York Heart Association
RV: Right ventricle/right ventricular
RVEDV: Right ventricular end-diastolic volume
RVEDVI: Right ventricular end-diastolic volume index
RVEF: Right ventricular ejection fraction
RVESV: Right ventricular end-systolic volume
RVESVI: Right ventricular end-systolic volume index
RVI: Right ventricular insertion
SCD: Sudden cardiac death
SCMR: Society for Cardiovascular Magnetic Resonance
