# Supplementary material for: Right ventricular insertion site fibrosis in a dilated cardiomyopathy referral population: phenotypic associations and value for the prediction of heart failure admission or death
Source: J Cardiovasc Magn Reson. 2021 Jun 17;23:79. doi: 10.1186/s12968-021-00761-0 (PMC8210339; doi:10.1186/s12968-021-00761-0)
Supplement: Supplementary file 1 — Additional file 1. Baseline clinical and CMR characteristics for patients with and without right ventricular insertion (RVI) site fibrosis. [file 12968_2021_761_MOESM1_ESM.docx]

**Additional file 1: Table S1.** Baseline clinical and CMR characteristics for patients with and without right ventricular insertion (RVI) fibrosis.

| **Characteristics** | **Total cohort (N=645)** | **RVI+ (N=274)** | **RVI- (N=371)** | **P value** |
| --- | --- | --- | --- | --- |
| Age (years) | 56 ± 14 | 57 ± 13 | 54 ± 14 | 0.045* |
| Female, n (%) | 180 (28) | 66 (24) | 114 (31) | 0.08 |
| Systolic blood pressure | 116 ± 18 | 114 ± 19 | 118 ± 17 | <0.001* |
| Diastolic blood pressure | 70 ± 13 | 69 ± 12 | 71 ± 13 | 0.03* |
| Heart rate | 71 ± 16 | 72 ± 16 | 70 ± 15 | 0.08 |
| BMI (kg/m^2^) | 29 ± 6 | 30 ± 7 | 29 ± 6 | 0.31 |
| Hypertension, n (%) | 228 (35) | 101 (37) | 127 (34) | 0.50 |
| Diabetes, n (%) | 128 (20) | 56 (20) | 72 (19) | 0.77 |
| Hyperlipidemia, n (%) | 343 (53) | 149 (54) | 194 (52) | 0.63 |
| Smoking, n (%) | 120 (20) | 51 (21) | 69 (20) | 0.91 |
| NYHA class III or IV, n (%) | 153 (26) | 80 (33) | 73 (21) | 0.002* |
| **Medications** | | | | |
| ACE-I or ARB, n (%) | 524 (81) | 241 (88) | 283 (76) | <0.001* |
| Beta blocker, n (%) | 530 (82) | 237 (87) | 293 (79) | 0.02* |
| Diuretics, n (%) | 331 (51) | 173 (63) | 158 (43) | <0.001* |
| Digoxin, n (%) | 56 (9) | 28 (10) | 28 (8) | 0.26 |
| Statin, n (%) | 253 (39) | 109 (40) | 144 (39) | 0.81 |
| CCB, n (%) | 76 (12) | 30 (11) | 46 (12) | 0.62 |
| Amiodarone, n (%) | 31 (5) | 16 (6) | 15 (4) | 0.35 |
| **CMR – Non LGE variables** |  |  |  |  |
| LVEDVI (ml/m^2^) | 113 ± 38 | 123 ± 40 | 106 ± 36 | <0.001* |
| LVESVI (ml/m^2^) | 74 ± 37 | 85± 39 | 66 ± 33 | <0.001* |
| LVEF (%) | 37 ± 11 | 33 ± 11 | 39 ± 10 | <0.001* |
| LV mass indexed (g/m^2^) | 70 ± 22 | 74 ± 23 | 66 ± 20 | <0.001* |
| RVEDVI (ml/m^2^) | 87 ± 25 | 92 ± 27 | 84 ± 22 | 0.002* |
| RVESVI (ml/m^2^) | 49 ± 22 | 54 ± 26 | 45 ± 17 | <0.001* |
| RVEF (%) | 46 ± 12 | 43 ± 13 | 48 ± 10 | <0.001* |
| LA volume indexed (ml/m^2^) | 43 ± 18 | 46 ± 19 | 40 ± 16 | <0.001* |

Continuous data are expressed as mean ± SD, categorical data as n (%). *p<0.05.

HR data were available for 644 patients, Smoking for 593 patients, NYHA for 584 patients, LV mass index for 640 patients, RVEDVI for 643 patients, RVESVI and RVEF for 642 patients and LA max indexed for 635 patients.

Abbreviations: EDVI End-Diastolic Volume Indexed to body surface area, EF Ejection Fraction, ESVI End-Systolic Volume indexed to body surface area, LA Left Atrial, LGE Late Gadolinium Enhancement, LV Left Ventricular, NYHA New York Heart Association, RV Right Ventricular
